# Skewed Differentiation of Circulating Vγ9Vδ2 T Lymphocytes in Melanoma and Impact on Clinical Outcome

**DOI:** 10.1371/journal.pone.0149570

**Published:** 2016-02-25

**Authors:** Francesca Toia, Simona Buccheri, Ampelio Anfosso, Francesco Moschella, Francesco Dieli, Serena Meraviglia, Adriana Cordova

**Affiliations:** 1 Department of Surgical, Oncological and Oral Sciences (DICHIRONS), University of Palermo, Palermo, Italy; 2 Department of Biopathology and Medical Biotechnologies (DIBIMED), University of Palermo, Palermo, Italy; 3 Central Laboratory of Advanced Diagnosis and Biomedical Research (CLADIBIOR), University of Palermo, Palermo, Italy; 4 Department for the Treatment and Study of Abdominal Diseases and Transplantation, Mediterranean Institute for Transplantation and Advanced Specialized Therapies (ISMETT), Palermo, Italy; Mie University Graduate School of Medicine, JAPAN

## Abstract

**Objective:**

The aim of this study was to evaluate over time circulating γδ T lymphocytes in melanoma patients in terms of frequency, effector functions, and relationship with clinical stage and evolution, by comparing preoperative values to those obtained at a mean follow-up of 36 months or in the event of recurrence or disease progression, and to those of healthy controls. Also, we correlated the presence of tumor-infiltrating γδ T lymphocytes with clinical evolution of melanoma.

**Results:**

Mean frequencies of circulating γδ T cells before and after melanoma removal were very similar and comparable to healthy subjects, but patients who progressed to stage III or IV showed a significantly decreased frequency of circulating Vγ9Vδ2 T cells. The distribution of Vγ9Vδ2 memory and effector subsets was similar in healthy subjects and melanoma patients at diagnosis, but circulating γδ T cells of patients after melanoma removal had a skewed terminally-differentiated effector memory phenotype. Highly suggestive of progressive differentiation toward a cytotoxic phenotype, Vγ9Vδ2T cells from patients at follow up had increased cytotoxic potential and limited cytokine production capability, while the opposite pattern was detected in Vγ9Vδ2T cells from patients before melanoma removal.

**Conclusions:**

Follow-up data also showed that tumor infiltrating γδ T cells were significantly associated with lower mortality and relapse rates, suggesting that they may serve as a prognostic biomarker, for human melanoma.

## Introduction

Malignant melanoma accounts for only 3% of cutaneous tumors, but shows high tendency to metastasize and is responsible for as much as 65% of death for skin cancers [1]. While the standard treatment for localized melanoma is surgical resection, the treatment of metastatic melanoma is still challenging and carries a significant treatment-related morbidity; advanced disease is still associated with a poor prognosis and further research is needed to provide more effective and tolerable therapeutic protocols for stage III and IV melanoma [2–4].

In the last 40 years, increasing interest has developed toward host cellular immune response and its possible therapeutic implication in the adjuvant therapy and advanced disease settings. Spontaneous regression of disease has been reported in patients with melanoma, suggesting a role for host immunity [5], which is indirectly supported by the evidence for lymphocyte infiltration of primary melanomas associated with tumor regression [6,7].

Tumor-infiltrating lymphocytes (TILs) have been shown to play an important role in the anti-tumor surveillance and have been documented in a wide variety of solid tumors including melanoma, breast, renal cell, prostate and colon cancers [8–10]. A potential prognostic and predictive significance has been suggested [11] and numerous clinical trials have focused on their possible use in adoptive immunotherapy [11–15]. CD4^+^ and CD8^+^ T cells are often reported as the predominant subset of lymphocytes within TILs [16–18] and currently represent a common target for adoptive immunotherapy [19,20]. However, increasing evidence exists for a role of γδ T lymphocytes in the anti-tumor surveillance in the periphery [21], which is supported by their localization within epithelia. γδ T lymphocytes can be distinguished into two subsets: those expressing the Vδ1 chain are a minor population in the peripheral blood but predominate in mucosal tissues [22], while the subset expressing the Vδ2 chain paired to the Vγ9 chain (here and after referred to as Vγ9Vδ2 T cells) predominates in peripheral blood and lymphoid tissues in human adults [22]. Vγ9Vδ2 T cells recognize non-peptidic antigens (phosphoantigens, PAgs) by a MHC-unrestricted mechanism [23,24] and typically perform anti-tumor immune responses (cytotoxicity, production of IFN-γ and TNF-α, and dendritic cell maturation) [25,26]. Thus, there is a substantial interest in γδ T cells in the context of immunotherapeutic strategies and several pilot studies have described a partial success of γδ T cell-based immunotherapy in different types of cancer after the application of aminobisphosphonates (n-BP) or PAgs plus IL-2 *in vivo* or after repetitive transfer of *in vitro* expanded Vγ9Vδ2 T cells [27,28].

In a recent study, we have demonstrated that γδ T lymphocytes are well represented amongst TILs in cutaneous melanomas, produce the pro-inflammatory cytokines TNF-α and IFN-γ and exert a strong cytotoxic activity against melanoma cells *in vitro* [29]. Strongly suggestive of their anti-tumor role, percentages of Vγ9Vδ2 T cells, but not total γδ or Vδ1 T cells, correlate with early stage of melanoma and absence of metastasis.

In this study, we have investigated the diagnostic and prognostic potential of a Vγ9Vδ2 T-cell-based blood analysis on the grounds, that it may provide a useful biomarker of the patient’s anti-tumor immune response. To this aim, we have evaluated circulating Vγ9Vδ2 T lymphocytes in terms of frequency, effector functions and relationship with clinical stage and evolution, in subjects before and after removal of melanoma, and compared them with healthy controls.

## Materials and Methods

### Characteristics of the study cohort and study design

In this study, we enrolled patients from the same cohort of patients of our previous study on cutaneous melanoma and γδ T lymphocytes [29]. The cohort consisted of 74 patients with histologically confirmed diagnosis of cutaneous melanoma, treated between March 2007 and March 2010 at the Plastic Surgery Unit of the University of Palermo. Forty-two patients were males and thirty-two were females; median age at surgery was 60 years (range 26–90). Sixty-nine patients (93%) presented with primary cutaneous melanoma and 5 patients with cutaneous or subcutaneous metastases (7%). All patients were staged according to the new American Joint Committee on Cancer staging system for cutaneous melanoma [30]. Patients were followed in a multidisciplinary melanoma clinic at the University of Palermo, and disease related mortality and relapse rates were recorded. Data were complemented with those obtained at a mean follow-up of 36 months. A blood sample was obtained from 38 out of 74 patients at the time of diagnosis and a second blood sample was obtained from 32 out of these 38 patients at the follow-up, or in the event of recurrence or disease progression. The clinicopathologic characteristics of the study cohort are given in Table 1. Blood samples from 45 age- and sex-matched healthy subjects in good and stable clinical condition, with laboratory parameters in the physiologic range, were used as a control.

**Table 1:** Clinicopathologic characteristics of the study cohort.

Characteristic	Patients N (%)
**Sex**	
Male	20 (62.5)
Female	12 (37.5)
**Tumor type**	
Primary melanoma	31 (96.9)
Cutaneous/subcutaneous metastasis	1 (3.1)
**Melanoma subtype**	
Superficial spreading	16 (50)
Nodular	11 (34.4)
Acrallentiginous	2 (6.3)
Spitzoid	3 (9.4)
**Localization**	
Head and neck	3 (9.4)
Trunk	15 (46.9)
Upper limb	3 (9.4)
Lower limb	11 (34–4)
**T Stage**	
Tis	3 (9.4)
T1	12 (37.5)
T2	9 (28.2)
T3	3 (9.4)
T4	5 (15.6)
**TNM Stage**	
0	3 (9.4)
I	14 (43.8)
II	11 (34.4)
**III**	3 (9.4)
**IV**	1 (3.1)

### Ethics Statement

The study was approved by the Ethical Committee of the University Hospital, Palermo, where the patients were recruited. The study was performed in accordance to the principles of the Helsinki declaration and those of the “Good Clinical Practices”, and all individuals gave written informed consent to participate.

### Isolation of PBMC and tumor-infiltrating immune cells, and FACS analysis

Peripheral blood mononuclear cells (PBMC) were obtained by density gradient centrifugation using Ficoll-Hypaque (Pharmacia Biotech, Uppsala, Sweden). Tissue specimens were obtained from 74 different patients undergoing standard-of-care surgical procedures for cutaneous melanoma, at the time of primary surgery. There were no restrictions (e.g. stage, etc) on tissues included for this study other than confirmation of melanoma by pathology review of H&E slides from the specimen taken for research. Tissue was obtained fresh and immediately transported to the laboratory in sterile saline for processing. Tissue was first minced into small pieces followed by digestion with collagenase type IV and DNAase (Sigma, St. Louis, MO) for 1 hr at 37°C. After digestion, the cells extracted were washed twice in RPMI 1640 medium (Gibco, Grand Island, NY, USA) and tumor-infiltrating mononuclear cells were obtained by Ficoll-Hypaque density gradient centrifugation. Both PBMC and tumor-infiltrating cells were stained for live/dead discrimination using Invitrogen LIVE/DEAD fixable violet dead cell stain kit (Invitrogen, Carlsbad, CA). Fc receptor blocking was performed with human immunoglobulin (Sigma, 3 μg/ml final concentration) followed by surface staining with different fluorochrome-conjugated antibodies to study the composition of the different subpopulations.

The fluorochrome-conjugated monoclonal antibodies (mAbs) used to characterized the entire population were the following: anti-CD45, anti-CD3, anti-CD14, anti-CD19, anti-pan γδ TCR, anti-Vδ1, anti-Vδ2, anti-CD27 and anti-CD45RA, all purchased from BD Biosciences (Mountain View, CA). Expression of surface markers was determined by flow cytometry on a FACSCanto II Flow Cytometer with the use of the FlowJo software (BD Biosciences). The cells were first gated for singlets (FSC-H *vs* FSC-A), then for lymphomonocytes (SSC-A *vs* FSC-A) and then for T lymphocytes (CD45 *vs* CD3). The T lymphocyte gate was further analyzed for uptake of the LIVE/DEAD stain to determine live *versus* dead cells and their expression of CD14 and CD19, taking only the live, healthy T cell population (L/D^-/low^,CD14^-^, CD19^-^). Pan-γδ TCR, Vδ1, Vδ2, CD27 and CD45RA cell surface expression was then determined from this gated population. For every sample at least 100.000 viable lymphomonocytes were acquired. Negative control (background) values were not subtracted, as the median backgrounds for isotype-matched mAbs was 0.0028% (range, 0.000%-0.0063%). Samples were considered positive if the number of cells was equal to or greater than 0.01% and at least 10 clustered events were apparent. This empiric cut-off value was predicted to be > 90% different from background, at an α of 0.05 [29,31].

### Flow cytometry-based γδ T cell functional assays

PBMC were resuspended at a concentration of 5x10^6^/ml in complete RPMI medium in the presence of Zoledronate (Novartis Pharma, Basel, Switzerland, 1 μM) or with PMA (BD Biosciences, 20 ng/ml) and ionomycin (BD Biosciences, 1 μM). Anti-CD107a/b mAb (BD Biosciences) was directly added to the culture at this time point [26]. Two hours after stimulation, Monensin (Sigma, 2 μM) was added and the cells were incubated overnight at 37°C in 5% CO_2_. Then PBMC were stained with anti-TCR Vδ2 and anti-CD3 mAbs for 15 min at 4°C. Samples were fixed, permeabilized according to manufacturer’s instructions and incubated with anti-IFN-γ (25723.11, BD Biosciences, Mountain View, CA), anti-perforin (δG9, Vinci-Biochem, Firenze, Italy) or anti-Granzyme B (GB11, BD Biosciences) mAbs or isotype-matched control mAbs, for 30 min at 4°C. After washing, the expression of surface CD107a/b and intracellular IFN-γ, perforin or Granzyme B was analyzed upon gating on the Vδ2^+^ T cell population.

### Statistical analysis

Statistical analysis was performed using the SPSS 20.0 software. The frequencies of circulating γδ T lymphocytes (preoperative values, follow-up values, healthy controls) were compared using nonparametric tests (Wilcoxon test, Mann Whitney test), based on the type of data and the sample size. Mortality and relapse rates in patients with and without tumor-infiltrating Vδ2 T lymphocytes at first diagnosis were compared using Chi squared test with Yates’ correction. Differences between groups with a p value ≤ 0.05 were regarded as significant.

## Results

### Ex vivo analysis of γδ T lymphocytes after melanoma removal

**Fig 1:**
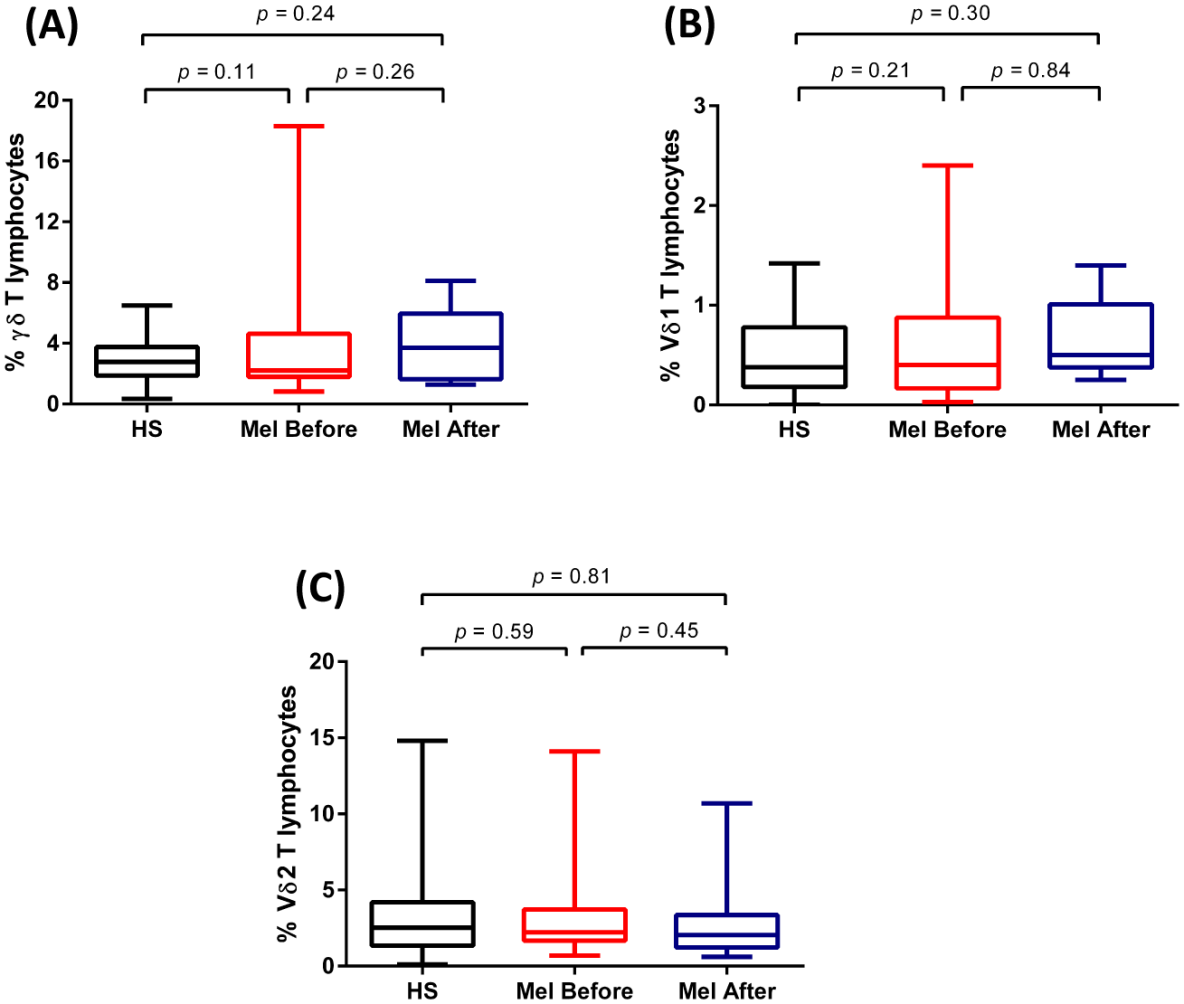
Percentages of total γδ T cells (A) and their Vδ1 (B) and Vγ9Vδ2 (C) subsets in healthy subjects and in melanoma patients before and after tumor removal. Box plots of percentages of γδ T cells subsets in 32 melanoma patients before and after melanoma removal and in 45 healthy subjects. Boxes represent 25th to 75th percentiles; middle bar identifies median; whiskers show minimum and maximum.

PBMC from 32 subjects were collected before surgical removal of a primary cutaneous melanoma and at a median follow-up of 36 months and were analyzed for the percentage of γδ T cells. The results were compared with those in a group of 45 sex- and age-matched healthy subjects (HS) used as control. As shown in Fig 1, the percentages of total γδ T cells (A) and their Vδ1 (B) and Vγ9Vδ2 (C) subsets were similar in melanoma patients before and after tumor removal and comparable to frequencies detected in HS, with no statistically significant differences between the three tested groups.

### Phenotypic and functional γδ T cell responses after melanoma removal

Human Vγ9Vδ2 T cells include those with naive or central-memory phenotypes (T_naive_, CD45RA^+^CD27^+^; T_CM_, CD45RA^-^CD27^+^) that home to secondary lymphoid organs, but that lack immediate effector function, and those with effector-memory (T_EM_, CD45RA^-^CD27^-^) and terminally-differentiated (T_EMRA_, CD45RA^+^CD27^-^) phenotypes that home to sites of inflammation and that display immediate effector functions as cytokine production and cytotoxic activity [32]. The phenotype and the functional responses of Vγ9Vδ2 T cells were analyzed before and at follow-up after melanoma removal and compared to those observed in healthy subjects.

**Fig 2:**
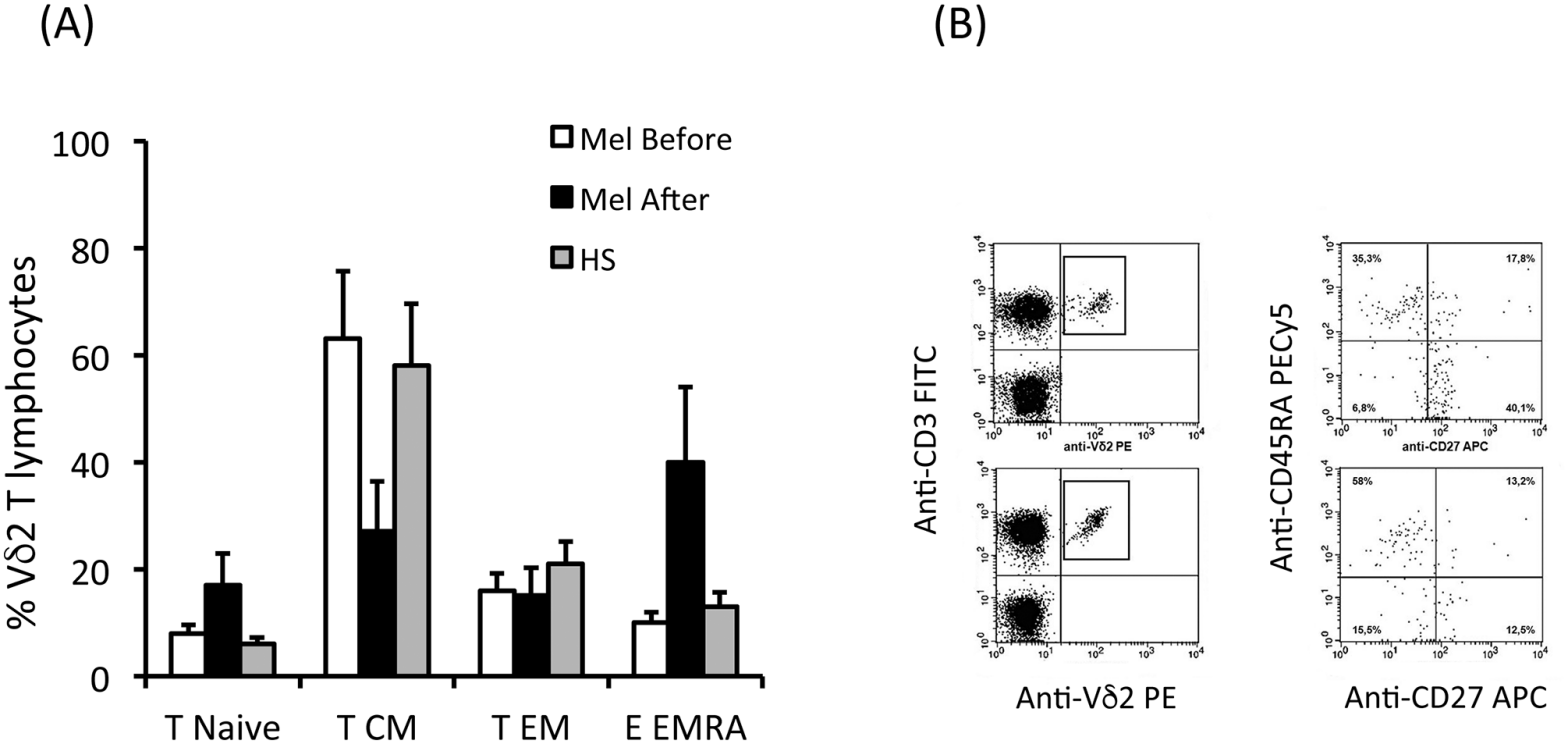
Phenotype of circulating Vγ9Vδ2 T cells before and after melanoma removal. PBMC were stained with anti-CD3, anti-Vδ2, anti-CD45RA and CD27 mAbs. Percentages of T_naive_ (CD45RA^+^CD27^+^), T_CM_ (CD45RA^-^CD27^+^), T_EM_ (CD45RA^-^CD27^-^) and T_EMRA_ (CD45RA^+^CD27^-^) cells were determined by FACS analysis. (A) Shows cumulative data for Vδ2 T cells and (B) shows representative flow cytometry panels from patient # 7 upon gating on CD3^+^Vδ2^+^ T cells and staining with CD27 and CD45RA.

As shown in Fig 2A, Vγ9Vδ2T cells obtained from PBMC of HS and melanoma patients before tumor removal showed a similar subset distribution with predominance of the T_CM_ (76.8% and 69.6%, respectively) and the T_EM_ (13% and 21%, respectively) phenotypes, which together accounted for approximately 90% of the whole Vγ9Vδ2 compartment. Conversely, T_Naive_ and T_EMRA_ subsets were very poorly represented amongst circulating Vγ9Vδ2 T cells of HS and patients before melanoma removal. As shown in Fig 2A, the majority of circulating Vγ9Vδ2 T lymphocytes in patients at follow up after melanoma removal expressed the T_EMRA_ phenotype, while the other three subsets were poorly represented.

This skewed phenotype of peripheral Vγ9Vδ2 T cells was strikingly depicted by the raw data for patient #7 shown in Fig 2B. Overall, the statistically significant changes in circulating Vγ9Vδ2T cell subset distribution indicate that a long-term effector maturation and mobilization of peripheral blood Vγ9Vδ2 T cells occur in subjects after melanoma removal and evoke previous observations after Zoledronate injection in cancer patients [33,34].

**Fig 3:**
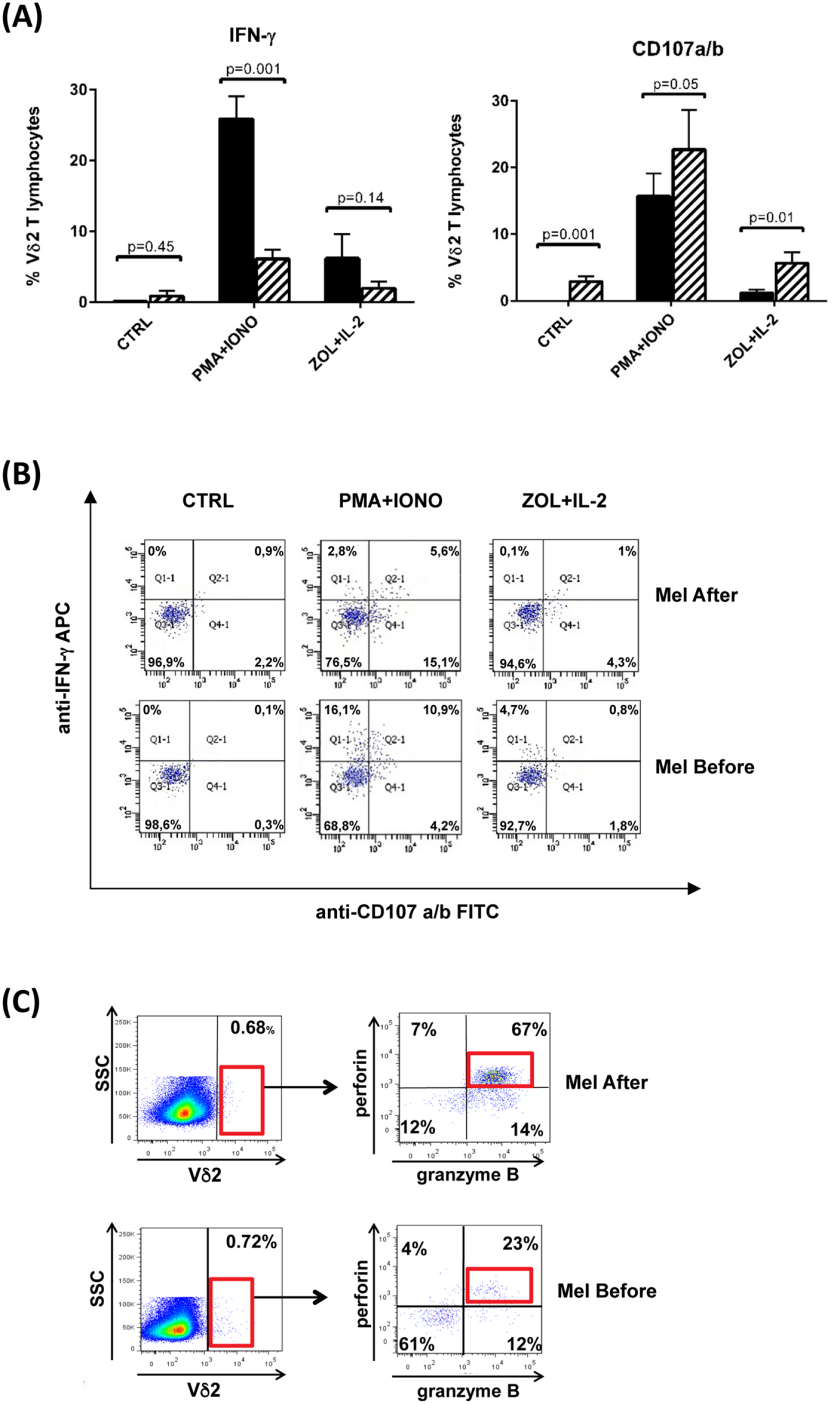
Functional responses of circulating Vγ9Vδ2 T cells in patients before and after melanoma removal. (A) Cumulative data and of intracellular IFN-γproduction and CD107a mobilization assays upon *in vitro* stimulation with Zoledronate or Iomomycin/PMA. (B) and (C) show raw data of intracellular IFN-γproduction and CD107a mobilization (B) and intracellular perforin and granzyme B expression by Vγ9Vδ2 of patient #21.

The phenotypic modifications of Vγ9Vδ2 T cells in subjects at follow up after melanoma removal were paralleled by modifications in their functional responses *in vitro*. Analysis performed in 14 patients showed (Fig 3A), that intracellular IFN-γ response to Iomomycin/PMA stimulation declined over time after melanoma removal, as compared to the response observed in the same patients before melanoma removal; IFN-γ response to Zoledronate stimulation also decreased after melanoma removal, but differences did not attain statistical significance. Conversely, the cytotoxic capability of Vγ9Vδ2 T cells (measured by the CD107 mobilization assay after Iomomycin/PMA or Zoledronate stimulation) consistently increased in patients after melanoma removal, and differences to values in patients before melanoma removal attained statistical significance. In accordance with the CD107a mobilization results, intracellular FACS analysis carried out on Vγ9Vδ2 T cells from 4 patients after melanoma removal revealed increased expression of perforin (70.3 ± 8,6% *versus* 25.3 ± 6.1%) and granzyme B (79.5 ± 10.4% *versus* 28.7 ± 6.7%). This trend is illustrated by raw data for patient #21 (Fig 3B and 3C).

### Correlation between circulating Vγ9Vδ2 T cells and clinical outcomes

**Fig 4:**
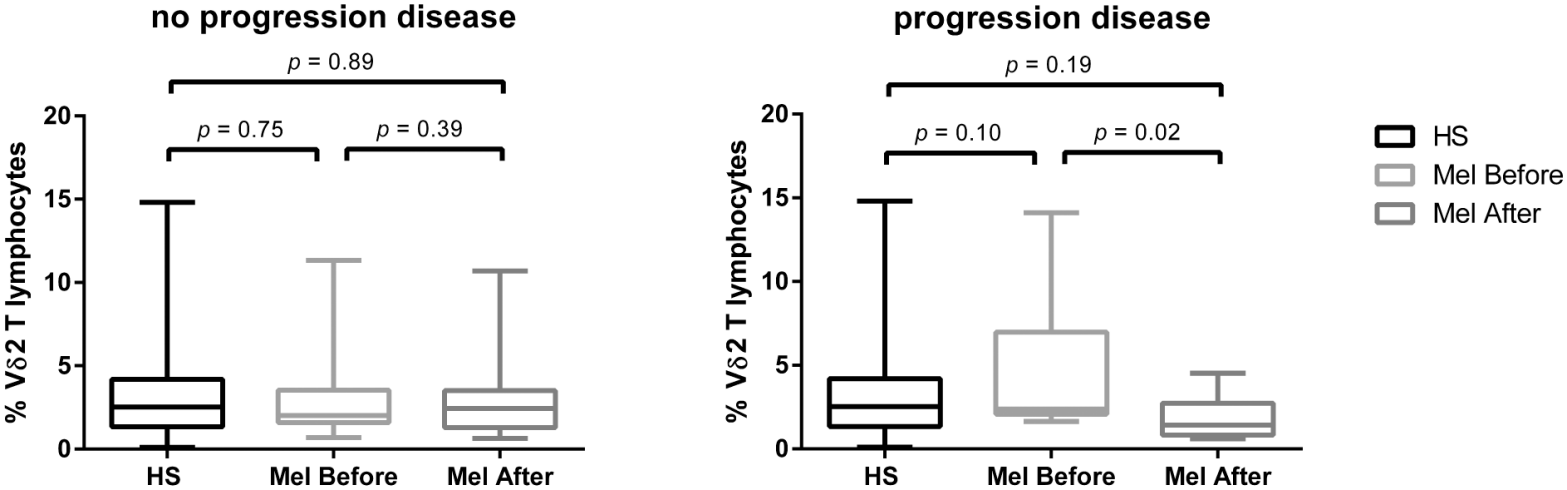
Percentages of Vγ9Vδ2 T cells in “progressor” and “not progressor” groups of melanoma patients and in healthy subjects. Boxes represent 25th to 75th percentiles; middle bar identifies median; whiskers show minimum and maximum.

Twelve out of the 32 studied patients relapsed and progressed after melanoma removal; six of them died before another blood sample was collected and thus follow-up data are available for only 6 patients, who progressed to stage III (1 patient) or stage IV (5 patients). Progression was invariably associated to substantial declines in Vγ9Vδ2 T cell numbers: as shown in Fig 4, patients who progressed to stage III or IV showed a decreased frequency of circulating Vγ9Vδ2 T cells at 36-months follow-up compared to values at diagnosis (4.55% to 1.81%). Conversely, patients who did not progress during the follow-up period showed a sustained or slightly increased frequency of circulating Vγ9Vδ2 T cells over time (2.94% to 3.15%). The Wilcoxon test for paired data showed a significant difference between preoperative and follow-up values in the “progressor” group of patients (p = 0.02), but not in the “non progressor” group (p = 0.39). Also, the Mann Whitney test showed a significant difference in the frequency variation over time between the “progressor” and “nonprogressor” groups (p = 0.01).

### Correlation of circulating and intratumoral Vγ9Vδ2 T cells with clinicopathological parameters of melanoma

To investigate further the clinical significance of Vγ9Vδ2 T cells in melanoma, the percentages of circulating and tumor-infiltrating Vγ9Vδ2 T cells at the time of diagnosis were analyzed relative to clinicopathological factors of melanoma patients, including cancer-specific survival rates, relapse-free survival (RFS) and overall survival (OS). We observed no statistically significant associations between the percentage of circulating Vγ9Vδ2 T cells before melanoma removal and stage, RFS and OS (data not shown). We then determined the impact of tumor-infiltrating Vγ9Vδ2 T cells on survival rates in a cohort of 74 previously studies melanoma patients [29] to determine the prognostic significance of intratumoral Vγ9Vδ2T cells. Previously, we reported that the presence of Vγ9Vδ2 T cells among tumor-infiltrating lymphocytes was positively correlated with earlier tumor stages (0, I and II). As shown in Table 2, the presence of Vγ9Vδ2 T cells among tumor-infiltrating lymphocytes was positively correlated not only with stage, but also with RFS and OS of melanoma patients: at a median follow-up of 36 months, the relapse rate was 13.0% when Vγ9Vδ2 T cells were present, and 35.7% in the absence of Vγ9Vδ2 T cells (Table 2). Similarly, the mortality rate was 21.4% in the absence of Vγ9Vδ2 T cell infiltration, but 0% when Vγ9Vδ2 T cells were present. The Chi squared test with Yates’ correction showed a significant difference between the “γδ positive” and “γδ negative” groups in both the mortality rate (p = 0.005) and the disease relapse rate (p = 0.045).

**Table 2:** Correlations between tumor-infiltrating Vγ9Vδ2 T cells and clinicopathologic characteristics in melanoma patients.

	Vγ9Vδ2 T cells amongst TILs		
Parameter	+ (n = 46)	- (n = 28)	p value
**Stage**			
0-I-II	68%	32%	0.035
III-IV	29%	71%	0.025
**Mortality rate**	0%	21.4%	0.005
**Relapse rate**	13%	35.7%	0.045

Mortality and relapse rate among 74 patients with cutaneous melanoma (median 36 months follow-up).

## Discussion

γδ T cells possess a combination of innate and adaptive immune cell qualities rendering them attractive for immunotherapy [21]. Two recent surveys [27,28] have reviewed the available published studies on the *in vivo* activation and adoptive transfer of *ex vivo*- expanded γδ T cells in cancer patients, providing evidence that Vγ9Vδ2 T cell-based immunotherapy improves overall survival and, in view of its low toxicity grade, provides a proof of principle for its utilization as adjuvant to conventional therapies for resistant/refractory patients care. Although γδ T cell-based immunotherapy has delivered promising results, several factor influence its success, amongst which is the finding of a low number and/or functionally unresponsive γδ T cells in patients with several types of tumors ([21,35]). For instance, Provinciali *et al*. [36,37] found that the number of circulating γδ T cells and their Vγ9Vδ2 subset was reduced in patients with melanoma and was not recovered after melanoma removal, as compared to the levels found in controls. However, Vγ9Vδ2 T cells had increased proliferative and cytokine-producing capability after melanoma removal, in comparison with the same subjects before surgical intervention or with control donors. Conversely, Petrini and coworkers [38] have found similar percentages of circulating γδ T cells in healthy control subjects and patients with melanoma, which had normal proliferative capacity but impaired cytotoxic activity, and Campillo *et al*. found increased frequencies of γδ T cells in patients with melanoma, which had high perforin content [39]

In this paper, we have evaluated the frequencies and functional properties of circulating Vγ9Vδ2 T lymphocytes in subjects before and after removal of melanoma, in relationship with clinical stage and evolution, and compared them with healthy controls. Data here reported clearly show that percentages of total γδ T cells and their Vδ1 and Vγ9Vδ2 subsets did not significantly differ in melanoma patients before and after tumor removal and in healthy control subjects. We do not have any explanation for the difference between our results and the previously reported γδ T cell studies in patients with melanoma. One possibility might be that changes in Vγ9Vδ2 T cell numbers and functions might be age- and sex-related and not a consequence of tumor growth. Accordingly, Vγ9Vδ2 T cells change characteristically with age, gradually decreasing beyond 30 years of age and drop more strikingly in men than in women [40]. Moreover, this loss is accompanied by a substantial depletion of Vγ9Vδ2 effector T cells, which is mirrored by a reduced IFN-γ secretion [40]. Accordingly, the absolute number of Vγ9Vδ2 T cells in a cohort of breast cancer patients receiving chemotherapy did not differ from age-matched breast cancer patients without treatment [41] and the decrease in absolute numbers of Vγ9Vδ2 T cells in a cohort of 41 patients with pancreatic ductal adenocarcinoma did not correlate with cancer stage/progression, but rather with patient age [42].

While we did not find any statistically significant difference in the distribution of Vγ9Vδ2 memory and effector subsets between healthy subjects and melanoma patients, lymphocytes expressing the T_EMRA_ phenotype were the dominant Vγ9Vδ2 T cell subset in patients at follow-up after melanoma removal. Highly suggestive of progressive differentiation toward a cytotoxic phenotype, Vγ9Vδ2 T cells from patients at follow-up had increased cytotoxic potential and limited cytokine production capability. Whether or not this predominant Vγ9Vδ2 T_EMRA_ phenotypic and functional status is associated with clinically relevant melanoma features is actually unknown. Intuitively, the accumulation of antigen-experienced Vγ9Vδ2 T_EMRA_ cells with a powerful cytotoxic potential could be considered beneficial in terms of disease control. However, recent studies in patients with chronic lymphatic leukemia have demonstrated that elevated numbers of circulating Vγ9Vδ2 T cells with dominant T_EM_ and T_EMRA_ phenotypes are a negative prognosticator [43], which is reminiscent of data previously reported in conventional CD4^+^ and CD8^+^ cells [44,45]; in these patients, T_EMRA_ CD8^+^ cells displayed the phenotypic hallmarks of functional exhaustion, further supporting the concept that long-lasting tumor-induced chronic activation may lead to the undesired accumulation of cells unable to exert effective antitumor activity. Indeed, several *in vitro* and *in vivo* data indicate that chronic Vγ9Vδ2 T cell activation may induce functional exhaustion or anergy. Anergy has been reported in HIV-infected individuals [46], whereas functional exhaustion has been described in a preclinical nonhuman primate model after repeated stimulations with PAgs and low-dose IL-2 [47] and has been associated with a differentiation shift, which is well in line with the accumulation of Vγ9Vδ2 T_EMRA_ cells.

In our study, we also evaluated the impact of baseline and follow-up Vγ9Vδ2 T cell counts on clinicopathological parameters of melanoma, to investigate whether these counts could be used as an alternative to predict outcome. While baseline percentages of circulating Vγ9Vδ2 T cells were not significantly correlated to any of the well established prognostic factors for melanoma (stage, RFS and OS), progression was invariably associated to substantial declines in Vγ9Vδ2 T cell numbers, reminiscent of our previous observations after Zoledronate and IL-2 injection in cancer patients.

We further determined prognostic significance of tumor-infiltrating Vγ9Vδ2T cells for the prediction of cancer development during the follow-up period in a cohort of 74 previously studies melanoma patients, and we show here that the presence of Vγ9Vδ2 T cells among tumor-infiltrating lymphocytes was positively correlated with RFS and OS of melanoma patients.

In this regard, our study, albeit small, emphasizes strongly a correlation of peripheral blood Vγ9Vδ2 T cells with arrested disease progression, and this evokes a comparable correlation of Vγ9Vδ2 T cell numbers (and most likely activities) with arrested disease progression in other scenarios of advanced malignancy [33,48]. We note, however, that the declining health of patients could not be attributed to poor functional conversion of Vγ9Vδ2 T cells (which was equivalent in all patients, data not shown). Therefore, the critical difference seems to be a failure to sustain robust Vγ9Vδ2 T cell numbers, implying that high response frequencies compose a key property of lymphoid stress-surveillance [8]. Hence, any future treatment optimizations should respect the need to achieve sufficiently large numbers of relevant Vγ9Vδ2 T cells.
